# The Effect of Transplanted Methylcholanthrene Induced Fibrosarcomata and Corynebacterium parvum on the Immune Response of CBA and A/HeJ Mice to Thymus Dependent and Independent Antigens

**DOI:** 10.1038/bjc.1974.2

**Published:** 1974-01

**Authors:** K. James, A. Ghaffar, I. Milne

## Abstract

The effect of transplanted syngeneic methylcholanthrene induced fibrosarcomata on the primary immune response of CBA and A/HeJ mice to standard doses of alum BSA, SRBC and SIII has been investigated. In animals with established fibrosarcomata the responses were (with one exception) either normal or elevated. Cell transfer studies in sublethally irradiated syngeneic recipients confirmed that the spleens from tumour bearing mice were capable of responding effectively to all 3 antigens. In animals simultaneously challenged with viable sarcoma cells and antigen the response to alum BSA was suppressed while those to SRBC and SIII were often enhanced. Furthermore the secondary response of A/HeJ mice to BSA was also suppressed by the simultaneous injection of viable fibrosarcoma cells. The administration of *C. parvum* 3 days after antigen had a variable effect. Nevertheless in a number of cases it significantly increased the primary response to all 3 antigens. It also inhibited the growth of the CBA fibrosarcoma but was without effect on the A/HeJ fibrosarcoma.


*Br. J. Cancer* (1974) **29**, 11

### THE EFFECT OF TRANSPLANTED METHYLCHOLANTHRENE INDUCED FIBROSARCOMATA AND *CORYNEBACTERIUM PARVUM* ON THE IMMUNE RESPONSE OF CBA AND A/HeJ MICE TO THYMUS DEPENDENT AND INDEPENDENT ANTIGENS

K. JAMES, A. GHAFFAR AND I. MILNE

*From the Department of Surgery, The University of Edinburgh Medical School, Teviot Place, Edinburgh EH8 9AG*

Received 9 August 1973. Accepted 3 September 1973

**Summary.—The effect of transplanted syngeneic methylcholanthrene induced fibrosarcomata on the primary immune response of CBA and A/HeJ mice to standard doses of alum BSA, SRBC and SIII has been investigated. In animals with established fibrosarcomata the responses were (with one exception) either normal or elevated. Cell transfer studies in sublethally irradiated syngeneic recipients confirmed that the spleens from tumour bearing mice were capable of responding effectively to all 3 antigens. In animals simultaneously challenged with viable sarcoma cells and antigen the response to alum BSA was suppressed while those to SRBC and SIII were often enhanced. Furthermore the secondary response of A/HeJ mice to BSA was also suppressed by the simultaneous injection of viable fibrosarcoma cells. The administration of *C. parvum* 3 days after antigen had a variable effect. Nevertheless in a number of cases it significantly increased the primary response to all 3 antigens. It also inhibited the growth of the CBA fibrosarcoma but was without effect on the A/HeJ fibrosarcoma.**

DURING the past decade there have been numerous investigations on the immune responsiveness of patients and animals with tumours of the lymphoreticular system or of other tissues. In general these studies indicate that there is an impairment of cell mediated immunity in many tumour bearing subjects including those with non-lymphomatous cancers. In contrast, the effect on humoral immunity is believed to be less marked (for example, see Miller, 1968; Southam, 1968).

As a result of recent advances in our understanding of humoral immune responses and the current interest in the use of adjuvants in tumour therapy, we felt it was necessary to determine the effect of established and simultaneously transplanted tumour on the humoral response to thymus dependent and independent antigens and to ascertain if this response could be modified by a *Corynebacterium parvum* (*C. parvum*) protocol which has previously been shown to inhibit tumour growth (Woodruff, Inchley and Dunbar, 1972). We have therefore studied the effect of transplanted syngeneic methylcholanthrene induced fibrosarcomata (MC fibrosarcomata) and this *C. parvum* schedule on the primary immune response of CBA and A/HeJ mice to bovine serum albumin (a thymus dependent antigen, Taylor, 1969), sheep erythrocytes (an essentially thymus dependent antigen, Playfair and Purves, 1971) and type III pneumococcus polysaccharide (a thymus independent antigen, Davies *et al.*, 1970; Howard *et al.*, 1971). As a further test of the immunocompetence of spleen cells from tumour bearing animals, we have also assessed their ability to restore the response of sublethally irradiated syngeneic recipients to thymus dependent and independent antigens.

### MATERIALS AND METHODS

*Mice*.—The experiments were performed in inbred adult (approximately 3-month old) male CBA/H or A/HeJ mice. The A/HeJ mice were bred by brother–sister mating from mice obtained from the Jackson Memorial Laboratories, Bar Harbor, Maine, U.S.A., while the CBA mice were bred from mice purchased from the M.R.C. Laboratory Animals Centre, Carshalton, Surrey.

*Tumours*.—The fibrosarcomata used were originally induced in 8–10 week old CBA and A/HeJ mice by a single intramuscular injection of 0·5 mg methylcholanthrene in 0·1 ml of trioctanoin. The tumours were propagated routinely by subcutaneous (s.c.) transplantation of a small piece of tissue. However, in the experiments to be described, transplantation was performed by the subcutaneous injection into the right thigh of a viable suspension prepared with pronase as previously described by Woodruff and Boak (1966). The CBA and A/HeJ fibrosarcomata had been transplanted 15 and 19–22 times respectively before experimentation. The tumour growths (expressed as mean diameter in mm) were continuously assessed throughout the period of experimentation.

*Corynebacterium parvum*.—This was a formalin killed suspension (Batch No. WEZ 174) kindly provided by Dr Griffiths of the Wellcome Research Laboratories, Langley Court, Beckenham, Kent. The suspension (containing 1·4 mg dry weight of organism) was routinely administered intraperitoneally (i.p.) 3 days following tumour transplantation. As previously indicated, this protocol has been shown to delay the growth of MC fibrosarcomata in CBA and A/HeJ mice (Woodruff *et al.*, 1972).

*Antigenic challenge and assessment of immune response*.—Details of the preparation of the alum precipitated bovine serum albumin (alum BSA) are recorded elsewhere (James and Milne, 1972). The mice were challenged i.p. with 0·2 ml of inoculum containing 1 mg of the protein and bled at selected time intervals thereafter. The antigen binding capacity (ABC) and relative binding affinity (RBA) of the sera were determined by the Farr procedure as previously described (Farr, 1958; James and Milne, 1972).

The sheep erythrocytes ($3 \times 10^8$ SRBC) were injected i.p. and 6 or 8 days later the direct and indirect plaque forming cell (PFC) response of individual spleens was determined by a modification of the Jerne plaque cell technique (Ghaffar and James, 1973*a*).

The purified type III pneumococcus polysaccharide antigen (SIII) was kindly supplied by Dr J. G. Howard of the Wellcome Research Laboratories, Beckenham, Kent. This was administered i.p. in 1 $\mu$g doses and the immune response elicited in individual spleens was again determined by the PFC technique 5 or 7 days following antigen challenge (Ghaffar and James, 1973*b*).

*Presentation of results*.—The ABC, PFC and tumour diameter data are all expressed as geometric mean values, together with the limits of one standard error from the mean. The RBA data are expressed as arithmetic means $\pm$ one standard error. Furthermore as the tumours and *C. parvum* sometimes caused splenomegaly, the PFC content per $10^6$ nucleated spleen cells and per spleen have both been presented. The significance of all the results (*P* values) have been determined by the standard 2 tail "*t*" test method. It should also be noted that the immune response to SRBC was also routinely assessed by standard serological procedures (Ghaffar and James, 1973*a*) but as these results were in close agreement with the PFC data they have been omitted in order to simplify presentation.

### RESULTS

### *The effect of transplanted fibrosarcoma cells and* C. parvum *on primary humoral responses*

In these experiments half the animals were challenged with i.p. injection of standard doses of one of the test antigens and at the same time they received a s.c. injection of $1 \times 10^4$ or $1 \times 10^5$ viable MC fibrosarcoma cells (see Tables I and II). The normal mouse controls were injected with antigen alone. Three days later half the mice in both the normal and

TABLE I.—*The Primary Immune Response to Thymus Dependent and Independent Antigens in CBA Mice Simultaneously Challenged with Viable Methylcholanthrene Induced Fibrosarcoma Cells* and Subsequently Treated with* Corynebacterium parvum

| Antigen | *C. parvum* | Assay | Normal mice controls† (mean ± s.e.) | Mice challenged with tumour (mean ± s.e.) | Significance‡ *P* |
|---|---|---|---|---|---|
| BSA (1 mg in alum) | No | Antigen binding capacity | 9·20 (8·82–9·60) | 5·75 (5·17–6·39) | 0·001 |
| | Yes | | 13·22 (11·1–15·75) | 6·48 (5·30–7·92) | 0·02 |
| | No | Relative binding affinity | 52·5 (47·1–56·9) | 50·4 (46·2–54·6) | 0·7 |
| | Yes | | 45·8 (41·9–49·6) | 61·8 (56·2–67·3) | 0·025 |
| SRBC ($3\times10^8$) | No | Direct PFC/$10^6$ | 20·8 (18–24) | 17·8 (15·5–20·5) | 0·4–0·5 |
| | | Direct PFC/spleen | 3223 (2856–3637) | 2523 (2236–2847) | 0·1–0·2 |
| | Yes | Direct PFC/$10^6$ | 18·2 (15·6–21·3) | 19·8 (16·4–23·8) | 0·7–0·8 |
| | | Direct PFC/spleen | 5148 (4293–6175) | 5046 (3170–6106) | 0·9–0·95 |
| | No | Indirect PFC/$10^6$ | 323 (254–412) | 286 (239–343) | 0·7 |
| | | Indirect PFC/spleen | 50,536 (37,325–68,420) | 39,828 (33,799–46,933) | 0·4–0·5 |
| | Yes | Indirect PFC/$10^6$ | 319 (258–396) | 381 (344–422) | 0·4–0·5 |
| | | Indirect PFC/spleen | 90,157 (69,400–117,120) | 97,224 (88,055–107,348) | 0·6–0·7 |
| SIII (1·0 μg) | No | Direct PFC/$10^6$ | 16·8 (12–23·5) | 19 (9–24) | 0·8–0·9 |
| | | Direct PFC/spleen | 1986 (1414–2789) | 2087 (1278–3409) | 0·9–0·95 |
| | Yes | Direct PFC/$10^6$ | 20 (15·8–25·5) | 37·3 (30·4–45·8) | 0·05–0·1 |
| | | Direct PFC/spleen | 3135 (2365–4154) | 7161 (5615–9133) | 0·05–0·1 |

* Animals injected with BSA received $1 \times 10^4$ viable cells, the others $1 \times 10^5$.
† Each group contained 4–8 mice.
‡ In all tables *P* refers to significance between tumour bearing or tumour challenged mice and "normal" controls.

TABLE II.—*The Primary Immune Response to Thymus Dependent and Independent Antigens in A/HeJ Mice Simultaneously Challenged with Viable Methylcholanthrene Induced Fibrosarcoma Cells* and Subsequently Challenged with* Corynebacterium parvum

| Antigen | *C. parvum* | Assay | Normal mice controls† (mean ± s.e.) | Mice challenged with tumour (mean ± s.e.) | Significance *P* |
|---|---|---|---|---|---|
| BSA (1 mg in alum) | No | Antigen binding capacity | 10·90 (9·82–12·11) | 4·12 (3·61–4·70) | <0·001 |
| | Yes | | 17·99 (15·05–21·51) | 14·55 (12·66–16·72) | 0·3–0·4 |
| | No | Relative binding affinity | 50·3 (46·0–54·6) | 50·5 (45·6–55·4) | 0·975 |
| | Yes | | 43·7 (40·7–46·7) | 59·8 (54·9–64·7) | 0·005 |
| SRBC ($3\times10^8$) | No | Direct PFC/$10^6$ | 23·3 (19·3–28·2) | 26·2 (25·1–27·4) | 0·5–0·6 |
| | | Direct PFC/spleen | 6757 (5841–7818) | 7012 (6173–7965) | 0·8–0·9 |
| | Yes | Direct PFC/$10^6$ | 14 (11·5–17) | 17·4 (14·7–20·7) | 0·4 |
| | | Direct PFC/spleen | 5231 (4812–5542) | 6936 (6060–7939) | 0·3 |
| | No | Indirect PFC/$10^6$ | 133·4 (120·3–145) | 127·4 (117–139) | 0·7–0·9 |
| | | Indirect PFC/spleen | 38,625 (36,456–40,924) | 37,193 (35,762–38,681) | 0·6 |
| | Yes | Indirect PFC/$10^6$ | 92 (73·6–115·6) | 149 (128–174) | 0·1 |
| | | Indirect PFC/spleen | 38,548 (26,813–44,513) | 59,332 (49,472–71,157) | 0·1–0·2 |
| SIII (1·0 μg) | No | Direct PFC/$10^6$ | 1·(0) (0·8–1·4) | 4·1 (3·1–5·4) | ≃0·005 |
| | | Direct PFC/spleen | 200 (145–275) | 721 (536–970) | 0·02 |
| | Yes | Direct PFC/$10^6$ | 2·(1) (1·6–2·8) | 2·8 (2·1–3·8) | ≃0·5 |
| | | Direct PFC/spleen | 639 (490–832) | 658 (480–901) | ≃0·95 |

* Animals injected with BSA received $1 \times 10^4$ viable cells, the others $1 \times 10^5$.

† Each group contained 6–10 mice.

tumour groups were injected i.p. with 1·4 mg *C. parvum*. The circulating antibody response to BSA was determined 21 days after challenge, while the splenic PFC response to SIII and SRBC were assessed 7 and 8 days respectively following antigen challenge. The results of these experiments are summarized in Tables I and II.

It will be observed that the simultaneous administration of $1 \times 10^4$ viable tumour cells along with alum BSA significantly suppressed the quantity of antibody elicited by this antigen (*i.e.* suppressed the ABC values). The administration of tumour cells, however, had no effect on the quality of the antibodies produced (RBA). Subsequent treatment with *C. parvum* potentiated the overall immune response to alum BSA in normal CBA mice but had no significant effect in tumour bearing animals (Table I). In contrast, it potentiated the immune response of both normal and tumour challenged A/HeJ mice (Table II). The *C. parvum* protocol used also significantly increased the relative binding affinity of antibody produced in tumour bearing animals.

On no occasion did the simultaneous administration of viable MC fibrosarcoma cells suppress the splenic PFC response to SRBC or SIII. In contrast, on one occasion the tumour cells appeared to potentiate the splenic PFC response (namely the response of A/HeJ mice to SIII—Table II).

The effect of the *C. parvum* therapy upon the response of normal and tumour bearing mice to SRBC was variable. On 3 out of 4 occasions it significantly increased the number of direct and indirect PFC per spleen in CBA mice. It should be noted that PFC per $10^6$ nucleated spleen cells were enhanced only on a few occasions. In contrast, the PFC response per spleen was potentiated only on one occasion in A/HeJ mice (Table III).

*C. parvum* treatment caused a definite increase in the anti-SIII response of both A/HeJ and CBA mice challenged with

TABLE III.—*The Primary Immune Response to Thymus Dependent and Independent Antigens in CBA Mice with Established Transplanted Methylcholanthrene Induced Fibrosarcomata**

| Antigen | Day† injected | Tumour size‡ (mm) | Assay | Day of assay† | Normal mice controls§ (mean ± s.e.) | Tumour bearing mice (mean ± s.e.) | Significance P |
|---|---|---|---|---|---|---|---|
| BSA (1 mg in alum) | 8 | 6·2 (5·9–6·6) | Antigen binding capacity | 23 | 2·75 (2·58–2·93) | 2·12 (1·93–2·33) | 0·025 |
| | | | Relative binding affinity | | 29·1 (27·8–30·4) | 28·6 (25·2–31·9) | 0·8–0·9 |
| SRBC ($3 \times 10^8$) | 14 | 11·4 (10·2–12·7) | Direct PFC/$10^6$ | 20 | 77 (68·3–87) | 71 (58–87·4) | 0·7–0·8 |
| | | | Direct PFC/spleen | | 9529 (8605–10,553) | 15,466 (12,089–19,755) | 0·10 |
| | | | Indirect PFC/$10^6$ | | 343 (312–378) | 339 (291–396) | 0·95 |
| | | | Indirect PFC/spleen | | 42,442 (38,538–46,743) | 74,064 (60,805–90,217) | 0·025 |
| SIII (1 μg) | 14 | 13·0 (12·1–14·0) | Direct PFC/$10^6$ | 19 | 9·8 (8·3–11·6) | 13·8 (10·5–18·2) | 0·30 |
| | | | Direct PFC/spleen | | 1527 (1279–1825) | 2901 (2194–3824) | 0·05 |

* $1 \times 10^5$ viable cells (s.c.) into right thigh.
† Day of tumour cell injection being Day 0.
‡ Mean tumour diameter (± s.e.) on day of antigen challenge.
§ Each group contained 9–10 mice.

tumour. However, it did not significantly affect this response in non-tumour bearing mice.

The effect of the *C. parvum* therapy on tumour growth also varied from strain to strain. In CBA mice the growth of the transplanted fibrosarcoma cells was significantly inhibited throughout the period of observation. The mean tumour diameter (together with the limits of one standard error from the mean) in *C. parvum* treated mice were 3·0 (1·2–5·3) and 17·0 (16·6–17·4) on Days 14 and 28 respectively. In animals not receiving *C. parvum* the corresponding diameters were 7·4 (7·0–7·8) and 20·4 (19·3–21·3). In contrast, *C. parvum* treatment had little effect on tumour growth in A/HeJ mice, the tumour diameters in the *C. parvum* treated animals being 12·6 (12·3–13·0) and 19·7 (19·0–20·4) on Days 14 and 28 respectively, while those in the non-parvum group were 13·4 (12·6–14·1) and 21·6 (21·0–22·2).

*The effects of established fibrosarcomata on primary humoral responses*

In order to determine the effect of established fibrosarcomata on the primary response to thymus dependent and independent antigens the following experiments were performed. Mice were injected s.c. with $1 \times 10^5$ viable fibrosarcoma cells on Day 0 and on Day 8 or 14, when the tumour was established, they were challenged with standard doses of the test antigens (see Tables III and IV). Antibodies to BSA were assayed 11–15 days following antigenic challenge while the splenic PFC responses to SIII and SRBC were determined 5 and 6 days respectively after challenge with these antigens.

With one exception (see CBA mice challenged with alum BSA—Table III) the presence of established tumour failed to suppress the primary immune response. Indeed, on occasions the primary response to SRBC and SIII was significantly

TABLE IV.—*The Primary Immune Response to Thymus Dependent and Independent Antigens in A/HeJ Mice with Established Transplanted Methylcholanthrene Induced Fibrosarcomata*

| Antigen | Day† injected | Tumour size‡ (mm) | Assay | Day of assay† | Normal mice controls | Tumour bearing mice | Significance *P* |
|---|---|---|---|---|---|---|---|
| BSA (1 mg in alum) | 8 | 6·7§ (6·4–6·9) | Antigen binding capacity | 23 | 5·46 (4·51–6·61) | 4·46 (3·99–4·98) | 0·4 |
| | | | Relative binding affinity | | 29·0 (25·3–32·7) | 31·1 (29·2–33·1) | 0·6–0·7 |
| | 14 | 20·2‖ (19·2–21·3) | Antigen binding capacity | 25 | 2·38 (2·03–2·80) | 2·84 (2·19–3·69)¶ | 0·5 |
| | | | Relative binding affinity | | Not determined | | |
| SRBC ($3 \times 10^8$) | 14 | 7·3 (7·1–7·5) | Direct PFC/$10^6$ | 19 | 45·8 (37–56·7) | 79·4 (69·6–90·6) | 0·05 |
| | | | Direct PFC/spleen | | 11,816 (9477–14,733) | 24,703 (20,734–29,433) | 0·01–0·02 |
| | | | Indirect PFC/$10^6$ | | 87 (61·4–123·7) | 367 (297–455) | 0·005–0·001 |
| | | | Indirect PFC/spleen | | 22,492 (15,649–32,326) | 110,519 (86,916–140,531) | 0·005–0·001 |
| SIII (1 μg) | 14 | 7·7 (7·4–7·9) | Direct PFC/$10^6$ | 20 | 3·2 (2·4–4·4) | 3·4 (2·5–4·5) | ≏0·9 |
| | | | Direct PFC/spleen | | 725 (545–965) | 849 (639–1126) | 0·70 |

* $1 \times 10^5$ viable cells (s.c.) into right thigh.
† Day of tumour cell injection being Day 0.
‡ Mean tumour diameter (± s.e.) on day of Ag challenge except for § and ‖ where the values are those observed 12 and 19 days respectively following tumour challenge.
¶ This group contained only 4 animals, all other groups contained 8–10.

potentiated in animals with pre-existing tumour, though this effect appeared to vary from strain to strain. For example, the response to SRBC was significantly increased in A/HeJ mice (Table IV) bearing tumours while the effect in CBA (Table III) mice was marginal. In contrast, the response to SIII was significantly enhanced in CBA (Table III) mice bearing tumours but not in A/HeJ mice (Table IV).

An examination of the growth of the established tumours in CBA mice treated with various antigens indicated that this growth was much slower in mice challenged with alum BSA than in other groups. For example, on Day 19 after tumour injection the tumour diameter in the alum BSA groups was 9·5 (8·4–10·8) while in the mice receiving SRBC and SIII the diameters were 13·5 (11·7–15·6) and 16·6 (14·9–18·5) respectively. In contrast, the growth of the established tumours in A/HeJ mice was similar in all groups.

*The ability of spleen cells from tumour bearing animals to restore humoral responsiveness to irradiated syngeneic recipients*

As a further measure of the immunological capacity of tumour bearing mice, lymphoid cells from A/HeJ mice challenged with $1 \times 10^5$ tumour cells 14 days previously were transferred into normal syngeneic animals. Following 600 rad whole body x-irradiation recipient mice were injected i.v. with $3{\cdot}5 \times 10^7$ viable nucleated spleen cells from either tumour bearing or normal donors. Two to 3 days after repopulation the animals were challenged i.p. with the standard doses of the 3 test antigens. Circulating antibodies to BSA were measured 18 days following challenge with this antigen while splenic PFC's were measured 5 and 6 days respectively following challenge with SIII and SRBC.

From the results summarized in Table V it can be seen that the response in mice repopulated with spleen cells from tumour bearing animals was almost identical to that observed in mice repopulated with spleen cells from normal animals.

*The effect of transplanted fibrosarcoma cells and* C. parvum *on secondary humoral responses*

A/HeJ mice were sensitized on Day 0 by the i.p. injection of 1 mg of alum BSA. On Day 21 half the mice were injected s.c. with $1 \times 10^5$ viable fibrosarcoma cells (the tumour challenged group) and 0·1 mg of soluble BSA (i.p.). The remainder (which served as controls) were challenged

TABLE V.—*The Ability of Sublethally X-irradiated* (600 *rad*) *A/HeJ Mice to Respond to Thymus Dependent and Independent Antigens Following Repopulation with Spleen Cells* from Normal and Tumour Bearing Mice*

| Antigen | Assay | X-irradiated mice† repopulated with syngeneic spleen cells from: Normal mice (mean + s.e.) | Tumour bearing mice (mean + s.e.) | Significance P |
|---|---|---|---|---|
| BSA (1 mg in alum) | Antigen binding capacity | 2·30 (1·73–3·06) | 2·33 (1·93–2·82) | 0·95 |
| SRBC ($3 \times 10^8$) | Direct PFC/$10^6$ | 4·4 (3·9–4·9) | 5 (4·4–5·7) | 0·4 |
| | Direct PFC/spleen | 817 (723–923) | 862 (727–1022) | 0·8 |
| | Indirect PFC/$10^6$ | 8·7 (4·8–15·8) | 8·6 (5·0–14·8) | 0·98 |
| | Indirect PFC/spleen | 1616 (869–3206) | 1464 (789–2717) | 0·95 |
| SIII (1 μg) | Direct PFC/$10^6$ | 21·3 (17–27) | 18·6 (13·5–25·7) | 0·7–0·8 |
| | Direct PFC/Spleen | 3464 (2643–4541) | 2624 (1892–3641) | 0·3 |

* Animals injected i.v. with $3{\cdot}5 \times 10^7$ viable spleen cells following x-irradiation and challenged with antigen 2–3 days later.
† Each group contained 4–10 mice.

TABLE VI.—*The Secondary Immune Response to Alum BSA* in A/HeJ Mice Simultaneously Challenged with Methylcholanthrene Induced Fibrosarcoma Cells and Subsequently Treated with* C. parvum

| *C. parvum* | Assay† | Normal mice controls‡ (mean ± s.e.) | Mice challenged with tumour (mean ± s.e.) | Significance *P* |
|---|---|---|---|---|
| No | Antigen binding capacity | 35·95 (32·8–39·4) | 26·31 (23·84–29·03) | 0·025–0·05 |
| | Relative binding affinity | 68·5 (63·1–73·9) | 59·3 (55·2–63·4) | 0·2 |
| Yes | Antigen binding capacity | 33·31 (29·52–37·59) | 18·66 (16·88–20·63) | 0·025 |
| | Relative binding affinity | 73·9 (69·0–78·7) | 67·5 (63·2–71·9) | >0·9 |

* Mice injected i.p. with 1 mg of alum BSA on Day 0 and challenged i.p. on Day 21 with 100 μg of soluble BSA with or without 1 × $10^5$ viable tumour cells s.c. *C. parvum* was administered on Day 24.
† The antibody assays were performed on Day 41, that is 20 days after secondary challenge.
‡ Each group contained 9–10 mice.

with antigen alone. Three days later (Day 24) half of the normal controls and half of the tumour challenged mice were injected i.p. with 1·4 mg of formalized *C. parvum*. On Day 41 antibody assays were performed on all the mice and the results obtained are summarized in Table VI.

It will be observed that the secondary ABC response in animals simultaneously injected with tumour cells is significantly lower than that in normal controls. In contrast, however, the tumour inoculation had no significant effect on the affinity of the antibodies produced. Furthermore, the *C. parvum* protocol used had no significant effect on secondary humoral immunity in tumour bearing or normal animals. In addition, it failed to inhibit the growth of the tumours significantly, the tumour diameter in *C. parvum* treated animals 17 days after transplantation being 10·4 (9·6–11·3) while that in untreated animals was 12·9 (11·8–14·2).

### DISCUSSION

From the results presented it can be concluded that in general there is no significant suppression of the primary humoral immune response to thymus dependent and independent antigens in CBA and A/HeJ mice with established transplanted MC fibrosarcomata. In contrast, the primary and secondary immune responses to alum BSA may be suppressed if viable fibrosarcoma cells are administered at the same time as the antigen. These results are in agreement with previous observations in animals (Mackay, 1964) and humans (Southam, 1968) with non-lymphomatous tumours. They contrast, however, with other observations in tumour bearing animals (Kamo and Ishida, 1971) and patients (Litton, Hughes and Fulthorpe, 1964; Lee, Rowley and Mackay, 1970). It is thus apparent that the effect of tumours on humoral immunity is a variable one and difficult to predict with any certainty.

Of particular interest in the present studies were the observations that preexisting tumour might potentiate the immune response to SRBC and SIII. A similar increase in the response to SRBC in sarcoma bearing rats (Alexander *et al.*, 1969) and to SIII antigen and diphtheria toxoid in a limited number of cancer patients (Leskowitz *et al.*, 1957) has been reported previously. It is appreciated that the increased splenic PFC responses reported in the present study are in many cases due largely to the splenomegaly associated with tumour growth.

The observations that *C. parvum* may potentiate the immune response of both normal and tumour bearing mice to thymus dependent and independent antigens have previously been noted by others in normal animals (Pinckard, Weir and McBride, 1967; Howard, Christie and Scott, 1973). In the present studies the enhanced responses observed were probably due largely to the splenomegaly associated with *C. parvum* treatment. It should be noted, however, that it has been claimed that *C. parvum* may suppress the response to SIII if administered at the same time as this antigen (Howard *et al.*, 1973).

The ability of *C. parvum* to delay the growth of transplanted MC induced fibrosarcomata in CBA mice and other murine tumours has been reported elsewhere (Woodruff *et al.*, 1972; Woodruff and Dunbar, 1973; Woodruff and Boak, 1966; Halpern *et al.*, 1966; Smith and Scott, 1973). Moreover, recent studies indicate that *C. parvum* can exert this effect in T deprived mice, suggesting that its antitumour effect depends primarily on macrophage stimulation (Woodruff, Dunbar and Ghaffar, 1973). The failure of *C. parvum* to delay the growth of MC fibrosarcomata in A/HeJ mice contrasts with the previous observations from this laboratory (Woodruff *et al.*, 1972). It should be noted, however, that the experimental models used in these experiments were different from those employed by the above authors in that an additional adjuvant was used in the present experiments. Of further interest are the observations that alum BSA injections significantly inhibited the growth of established fibrosarcomata in CBA mice, but not in A/HeJ mice. This was probably due to the nonspecific stimulation of the reticuloendothelial system by the alum.

In Table VII the overall effect of our *C. parvum* protocol on humoral immunity in tumour bearing animals has been summarized. This suggests that the *C. parvum* is more effective at potentiating 19*s* (IgM) responses in CBA mice with fibrosarcomata than in A/HeJ mice with similar tumours. It is possible therefore that its superior tumour inhibiting potential in the CBA mice was due partly to its capacity to elicit antitumour antibodies of the IgM class, for these are generally held to be more effective at lysing target cells than their 7*s* (IgG) counterparts. Conversely the ineffectiveness of the *C. parvum* protocol in A/HeJ mice may be due to its inability to influence significantly the levels of cytotoxic IgM antibodies in this strain, or alternatively because it favoured the production of blocking antibodies.

At the present time we have no satis-

TABLE VII.—*A Summary of the Effect of the* Corynebacterium parvum *Protocol on the Humoral Immune Response in Mice Simultaneously Transplanted with Viable Fibrosarcoma Cells*

| Mouse strain | Antigen | Assay | Effect | Main antibody class |
|---|---|---|---|---|
| CBA | Alum BSA | ABC | No effect | IgG |
| | SRBC | Direct PFC | Significant potentiation | IgM |
| | | Indirect PFC | Significant potentiation | IgG |
| | SIII | Direct PFC | Significant potentiation | IgM |
| | Tumour | Growth | Significant inhibition | |
| A/HeJ | Alum BSA | ABC | Significant potentiation | IgG |
| | SRBC | Direct PFC | No significant effect | IgM |
| | | Indirect PFC | Significant potentiation | IgG |
| | SIII | Direct PFC | No significant effect | IgM |
| | Tumour | Growth | No significant effect | |

Note: In tumour bearing CBA mice the *C. parvum* potentiated the formation of IgM antibody but failed to do so in A/HeJ mice.

factory explanation of the differing effects of established fibrosarcomata and of simultaneously administered viable tumour cells on the immune response to alum BSA and the other antigens under test. It is possible that during the initial growth phase of the tumour there is a tumour dependent depression of T cell function. As a result the immune response to antigens such as BSA, with a high degree of thymus dependency, may be suppressed. On the other hand, the response to antigens such as SRBC (which are less dependent upon T cell helper function), and SIII (which does not require a T cell helper effect) are not significantly affected. Later, when the tumour has become established, there is a recovery in T cell function enabling the animals to mount a normal response to alum BSA. In this connection it is interesting to note that a humoral factor which depresses cell mediated immunity has recently been reported in patients with primary intracranial tumours (Brooks *et al.*, 1972).

While the present studies clearly indicate that the overall immune response to fixed doses of thymus dependent and independent antigens is not affected by established MC fibrosarcomata in CBA and A/HeJ mice, they are of themselves limited. It is possible that tumours exert a local immune suppressive effect in draining lymph nodes and this would not be detected in our assays. Furthermore, it is also feasible that the dose of thymus dependent antigens used was sufficient to overcome any deficiency in T cell function (Sinclair and Elliott, 1968; Taylor and Wortis, 1968). Finally, it is also appreciated that the effect of established tumours on humoral immune response may be dependent upon the type of tumour, its location and its host.

The authors wish to thank Professor M. F. A. Woodruff for his continuing advice and encouragement, and J. Merriman for his valuable technical assistance. They are also greatly indebted to the Cancer Research Campaign for generous financial assistance.

### REFERENCES

Alexander, P., Bensted, J., Delmore, E. J., Hall, J. G. & Hodgett, J. (1969) The Cellular Immune Response to Primary Sarcomata in Rats. II. Abnormal Responses of Nodes Draining the Tumour. *Proc. R. Soc. Lond. B*, **174**, 237.

Brooks, W. H., Netsky, M. G., Normansell, D. E. & Horwitz, D. A. (1972) Depressed Cell Mediated Immunity in Patients with Primary Intracranial Tumours. Characterization of a Humoral Immunosuppressive Factor. *J. exp. Med.*, **136**, 1631.

Davies, A. J. S., Carter, R. L., Leuchars, E., Wallis, V. & Dietrich, F. M. (1970) The Morphology of Immune Reactions in Normal, Thymectomized and Reconstituted Mice. III. Response to Bacterial Antigens: Salmonellar Flagellar Antigen and Pneumococcal Polysaccharide. *Immunology*, **24**, 454.

Farr, R. S. (1958) A Quantitative Immunochemical Measure of the Primary Interaction between $^{131}$I-labelled Bovine Serum Albumin and Antibody. *J. infect. Dis.*, **103**, 239.

Ghaffar, A. & James, K. (1973*a*) The Effect of Antilymphocytic Antibody on the Humoral Immune Response in Different Strains of Mice. II. The Response to Sheep Erythrocytes. *Immunology*, **24**, 454.

Ghaffar, A. & James, K. (1973*b*) The Effect of Antilymphocytic Antibody on the Humoral Immune Response in Different Strains of Mice. III. The Response to Type III Pneumococcus Polysaccharide. *Immunology*, **24**, 1075.

Halpern, B. N., Biozzi, G., Stiffel, C. & Mouton, D. (1966) Inhibition of Tumour Growth by Administration of Killed *Corynebacterium parvum*. *Nature, Lond.*, **212**, 853.

Howard, J. G., Christie, G. H., Courtenay, B. M., Leuchars, E. & Davies, A. J. S. (1971) Studies on Immunological Paralysis. VI. Thymic Independence of Tolerance and Immunity to Type III Pneumococcal Polysaccharide. *Cell. Immun.*, **2**, 614.

Howard, J. G., Christie, G. H. & Scott, M. T. (1973) Biological Effects of *Corynebacterium parvum*. IV. Adjuvant and Inhibitory Activities on B lymphocytes. In Ciba Foundation Symposium on *Immunopotentiation*, No. 18 (new series). In the press.

James, K. & Milne, I. (1972) The Effect of Antilymphocytic Antibody on the Humoral Immune Response in Different Strains of Mice. I. The Response to Bovine Serum Albumin. *Immunology*, **23**, 897.

Kamo, I. & Ishida, N. (1971) Immune Response in Tumour Bearing Mice. *Gann*, **62**, 453.

Lee, A. K. Y., Rowley, M. & Mackay, I. R. (1970) Antibody Response in Human Cancer. *Br. J. Cancer*, **24**, 454.

Leskowitz, S., Phillipino, L., Hendrick, G. & Graham, J. B. (1957) Immune Response in Patients with Cancer. *Cancer, N.Y.*, **10**, 1103.

Litton, B., Hughes, L. E. & Fulthorpe, A. J. (1964) Circulating Antibody Response in Malignant Disease. *Lancet*, i, 69.

MACKAY, W. D. (1964) Antibody Production in Mice with Isologous Tumours. *Nature, Lond.*, **207**, 1306.

MILLER, D. G. (1968) The Immunologic Capability of Patients with Lymphoma. *Cancer Res.*, **28**, 1441.

PINCKARD, R. N., WEIR, D. M. & MCBRIDE, W. H. (1967) Factors Influencing the Immune Response. I. Effects of the Physical State of the Antigen and of Lymphoreticular Cell Proliferation on the Response to Intravenous injection of Bovine Serum Albumin in Rabbits. *Clin. & exp. Immunol.*, **2**, 331.

PLAYFAIR, J. H. L. & PURVES, E. C. (1971) Antibody Formation by Bone Marrow Cells in Irradiated Mice. I. Thymus Dependent and Thymus Independent Responses to Sheep Erythrocytes. *Immunology*, **21**, 113.

SINCLAIR, N. R. ST C. & ELLIOT, E. V. (1968) Neonatal Thymectomy and Decrease in Antigen Sensitivity of the Primary Response and Immunological Memory Systems. *Immunology*, **15**, 325.

SOUTHAM, C. M. (1968) The Immunological Status of Patients with Non-lymphomatous Cancer. *Cancer Res.*, **28**, 1433.

SMITH, S. E. & SCOTT, M. T. (1972) Biological Effects of *Corynebacterium parvum*. III. Amplication of Resistance and Impairment of Active Immunity to Murine Tumours. *Br. J. Cancer.*, **26**, 361.

TAYLOR, R. B. (1969) Cellular Cooperation in the Antibody Response of Mice to Two Serum Albumins: Specific Function of Thymus Cells. *Transplantn Rev.*, **1**, 114.

TAYLOR, R. B. & WORTIS, H. H. (1968) Thymus Dependence of Antibody Response: Variation with Dose of Antigen and Class of Antibody. *Nature, Lond.*, **220**, 927.

WOODRUFF, M. F. A. & BOAK, J. L. (1966) Inhibitory Effect of Injection of *C. parvum* on the Growth of Tumour Transplants in Isogeneic Hosts. *Br. J. Cancer*, **20**, 345.

WOODRUFF, M. F. A., INCHLEY, M. P. & DUNBAR, N. (1972) Further Observations on the Effect of *C. parvum* and Antitumour Globulin on Syngeneically Transplanted Mouse Tumours. *Br. J. Cancer*, **26**, 67.

WOODRUFF, M. F. A. & DUNBAR, N. (1973) The Effect of *Corynebacterium parvum* and Other Reticuloendothelial Stimulants on Transplanted Tumours in Mice. In Ciba Foundation Symposium on *Immunopotentiation*, No. 18 (new series). In the press.

WOODRUFF, M. F. A., DUNBAR, N. & GHAFFAR, A. (1973) The Growth of Tumours in T-cell Deprived Mice and their Response to Treatment with *Corynebacterium parvum*. *Proc. R. Soc., Lond.*, **184**, 97.


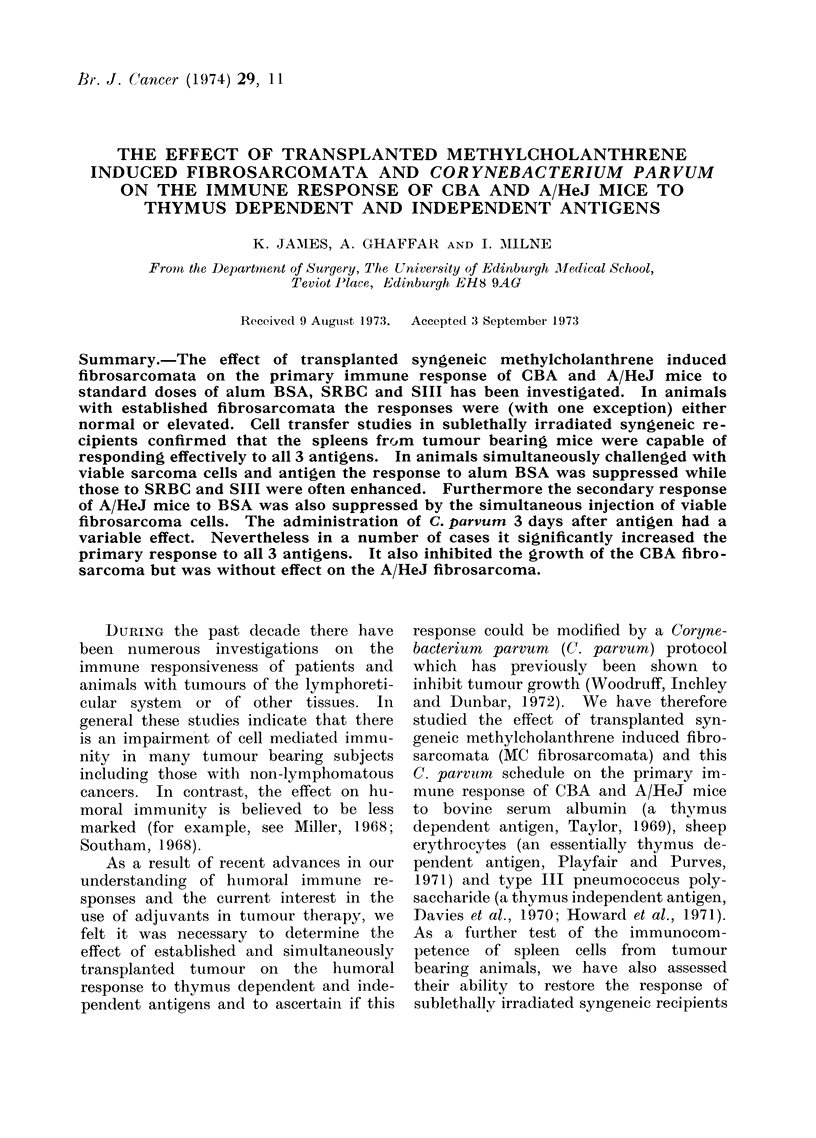

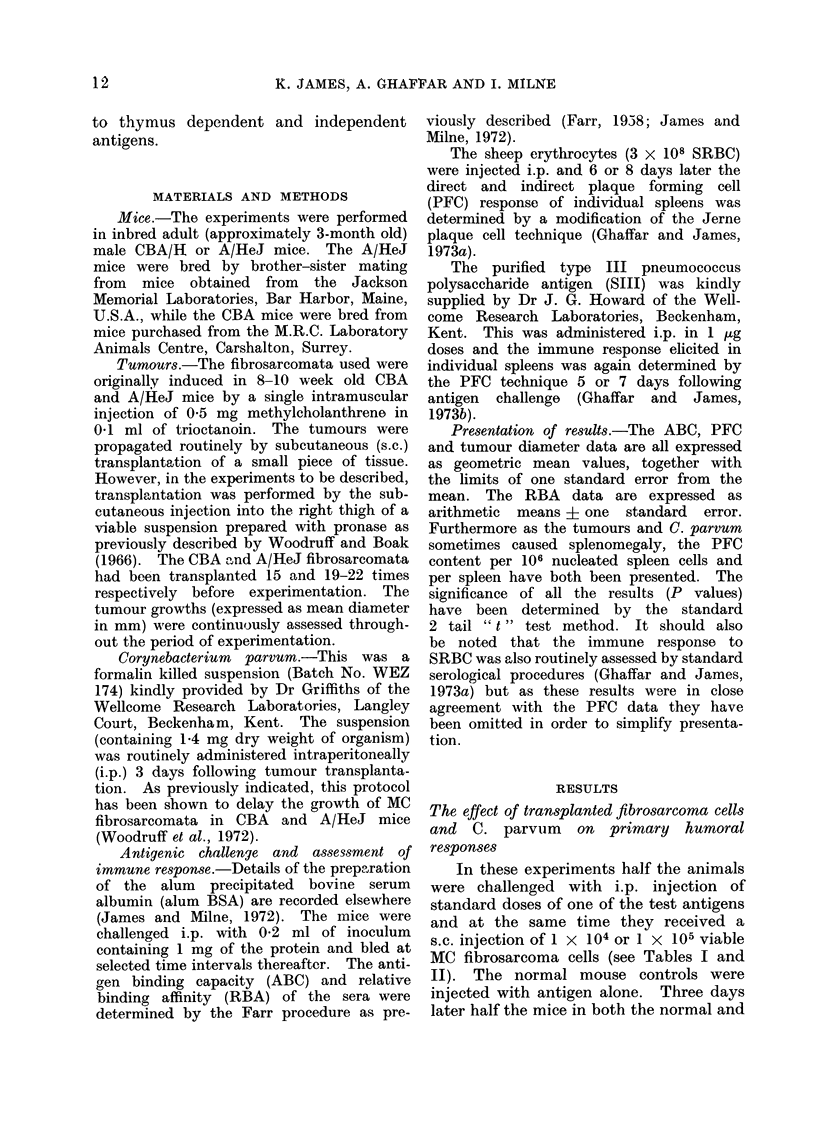

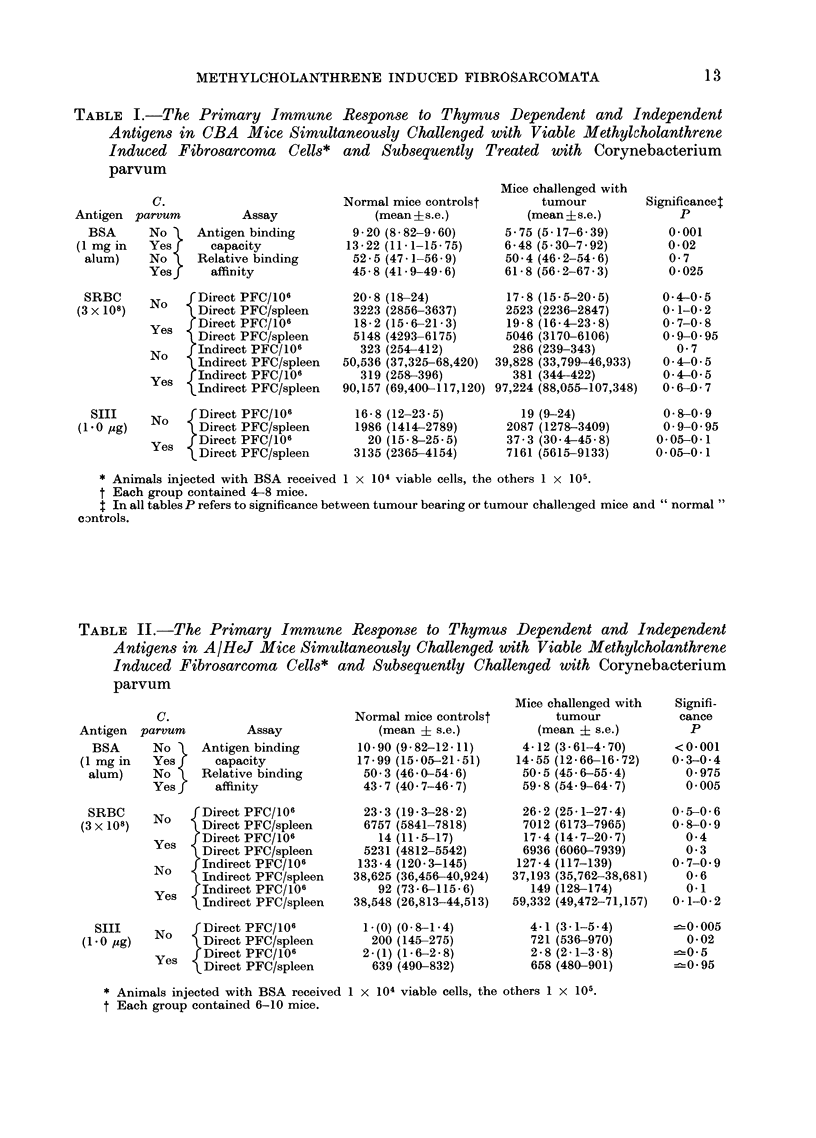

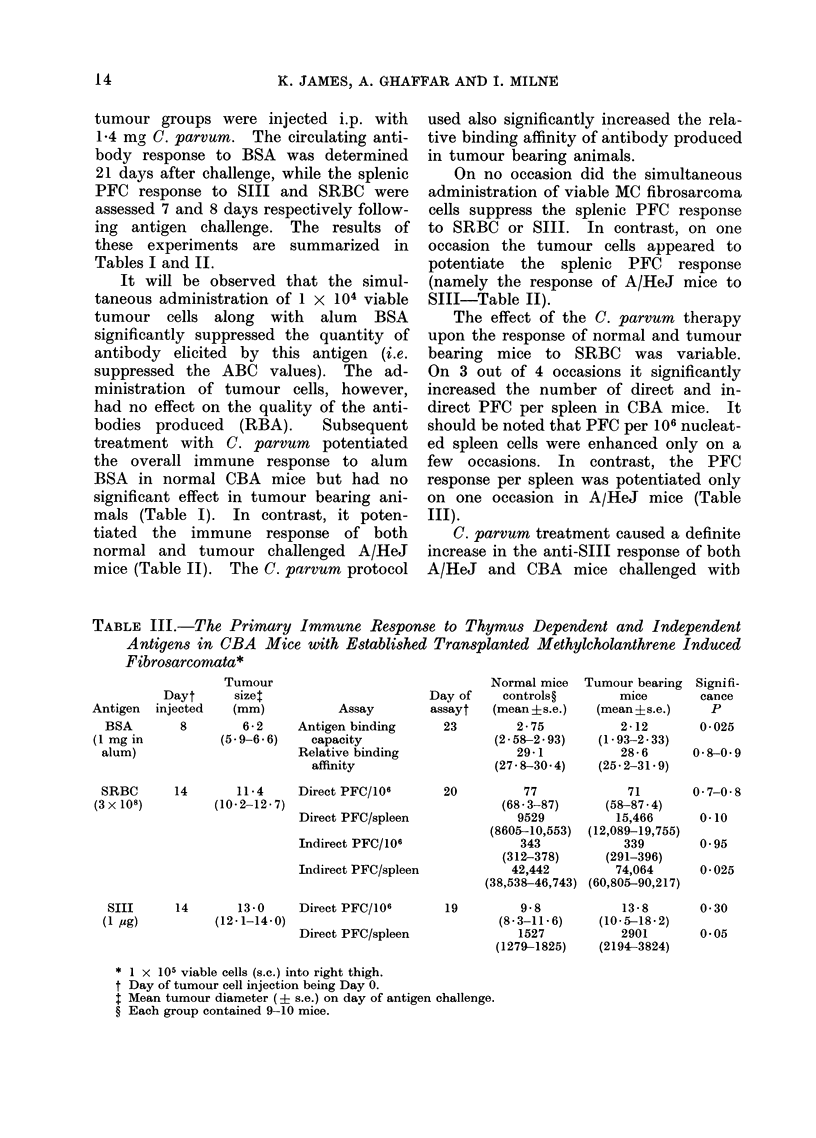

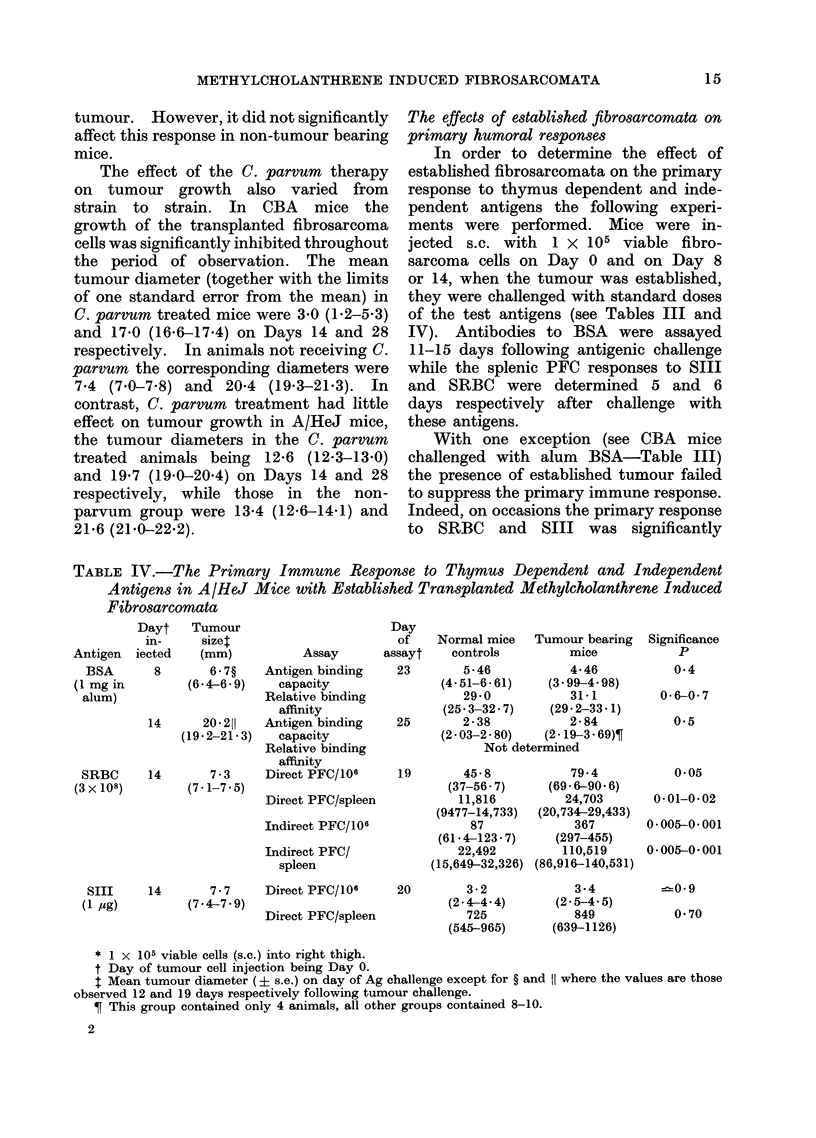

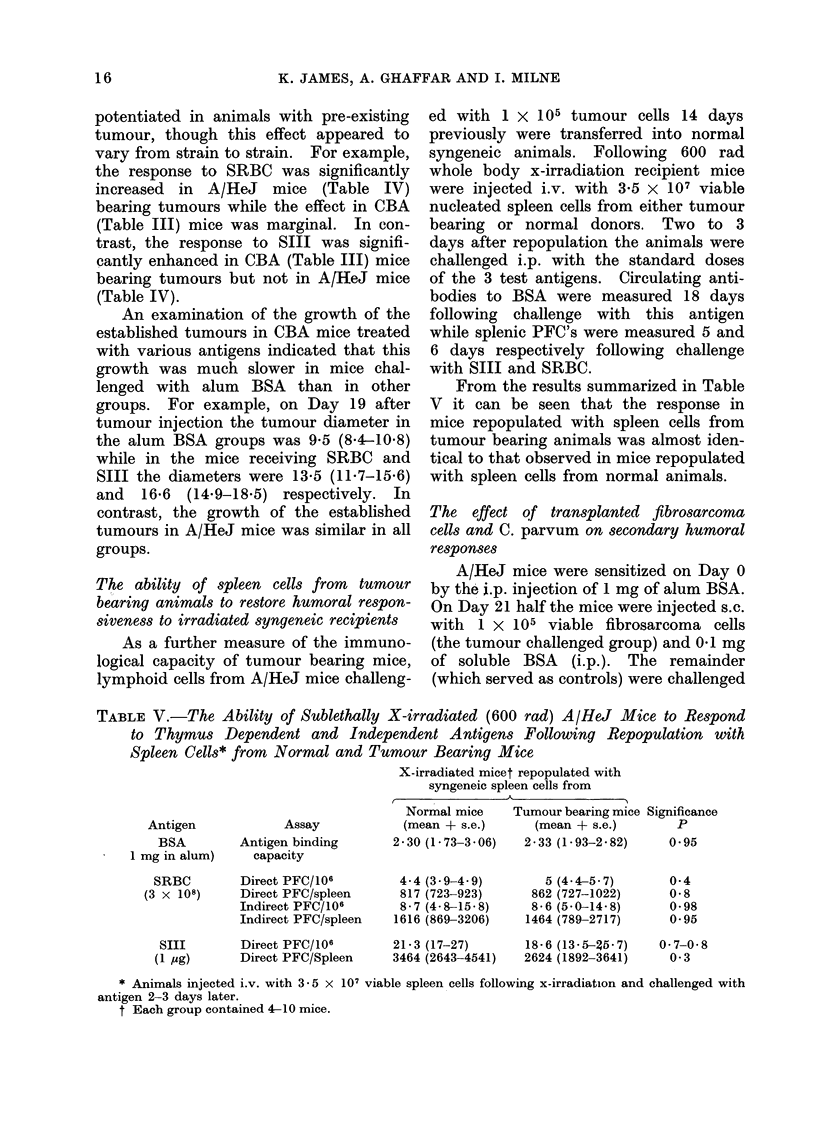

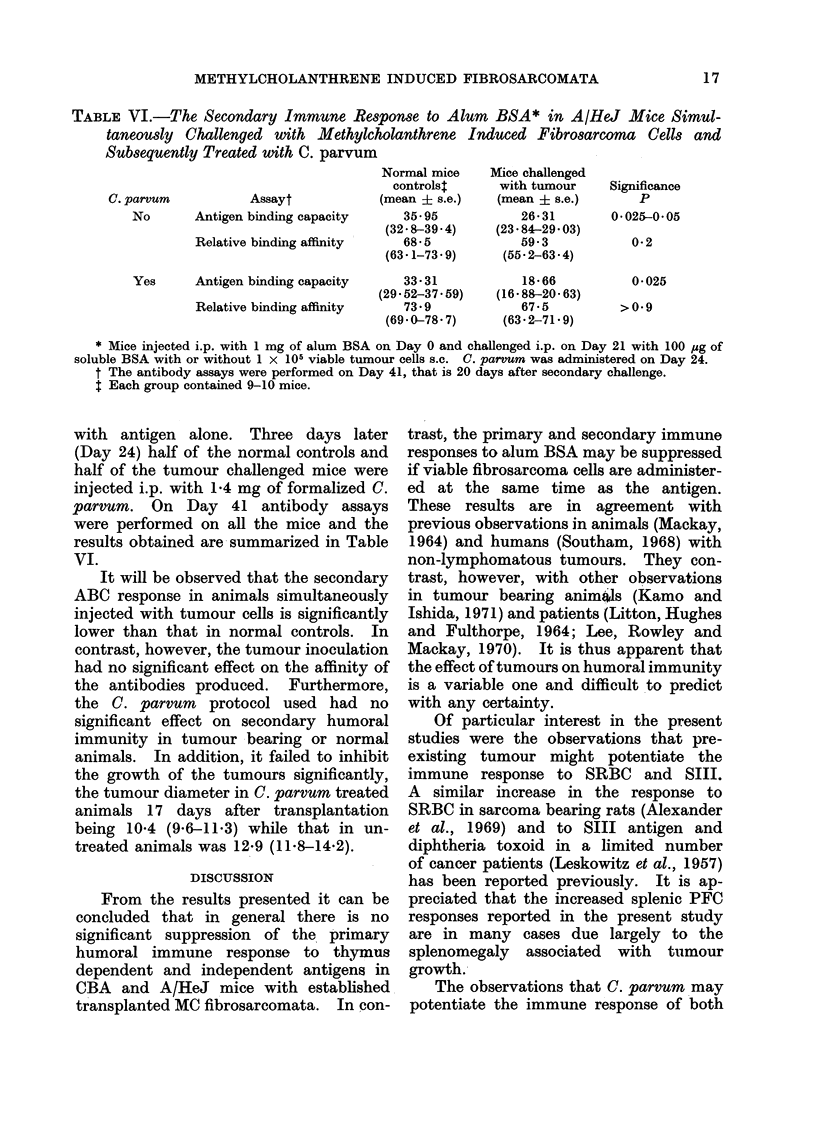

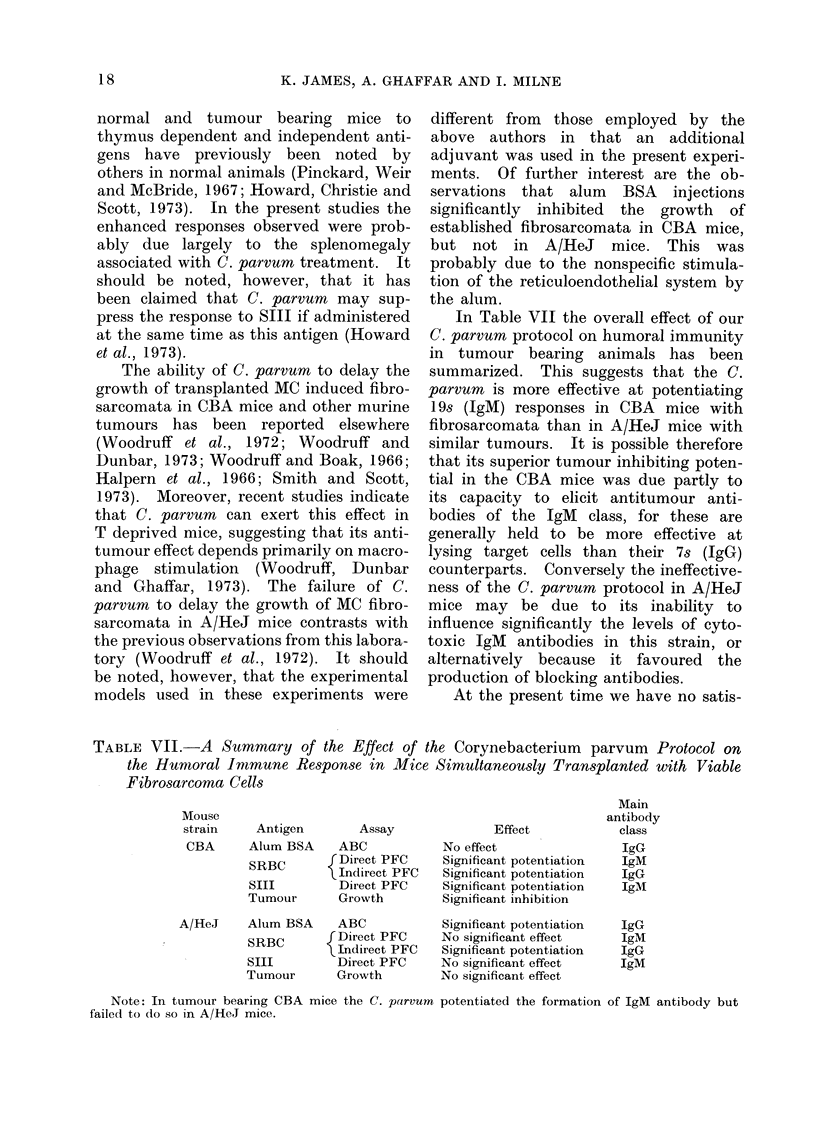

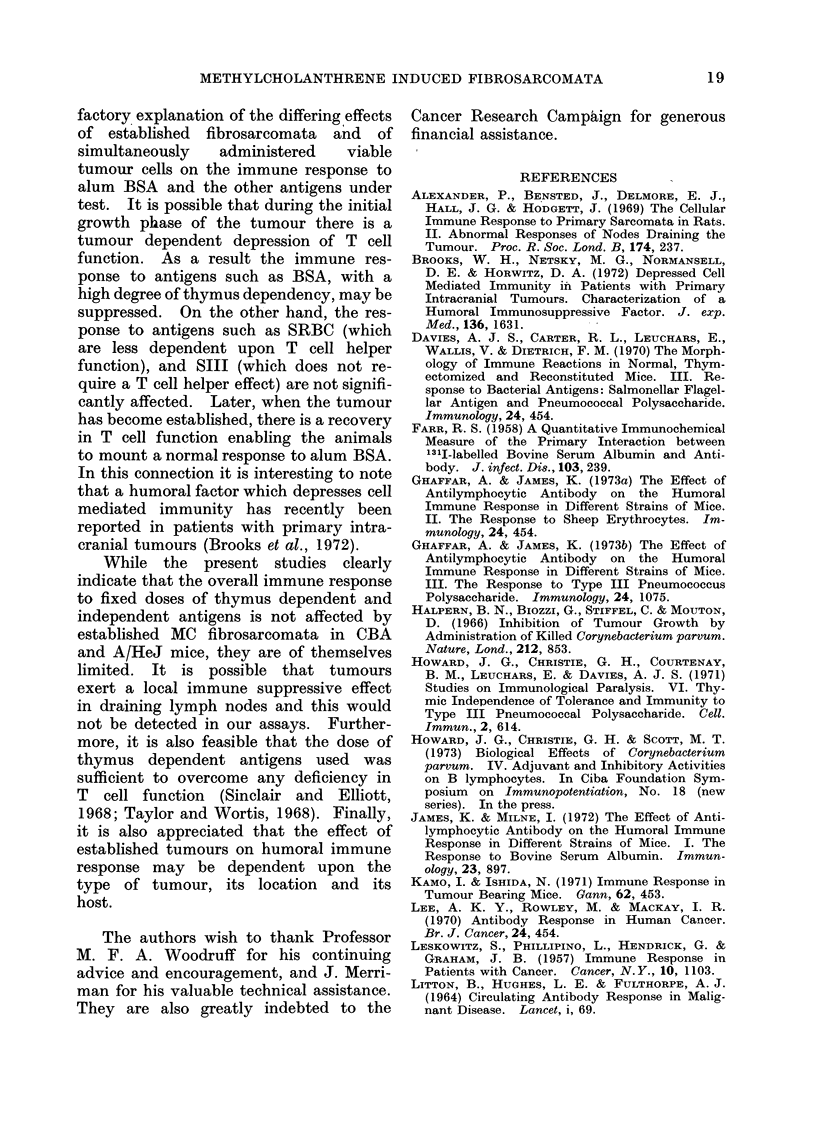

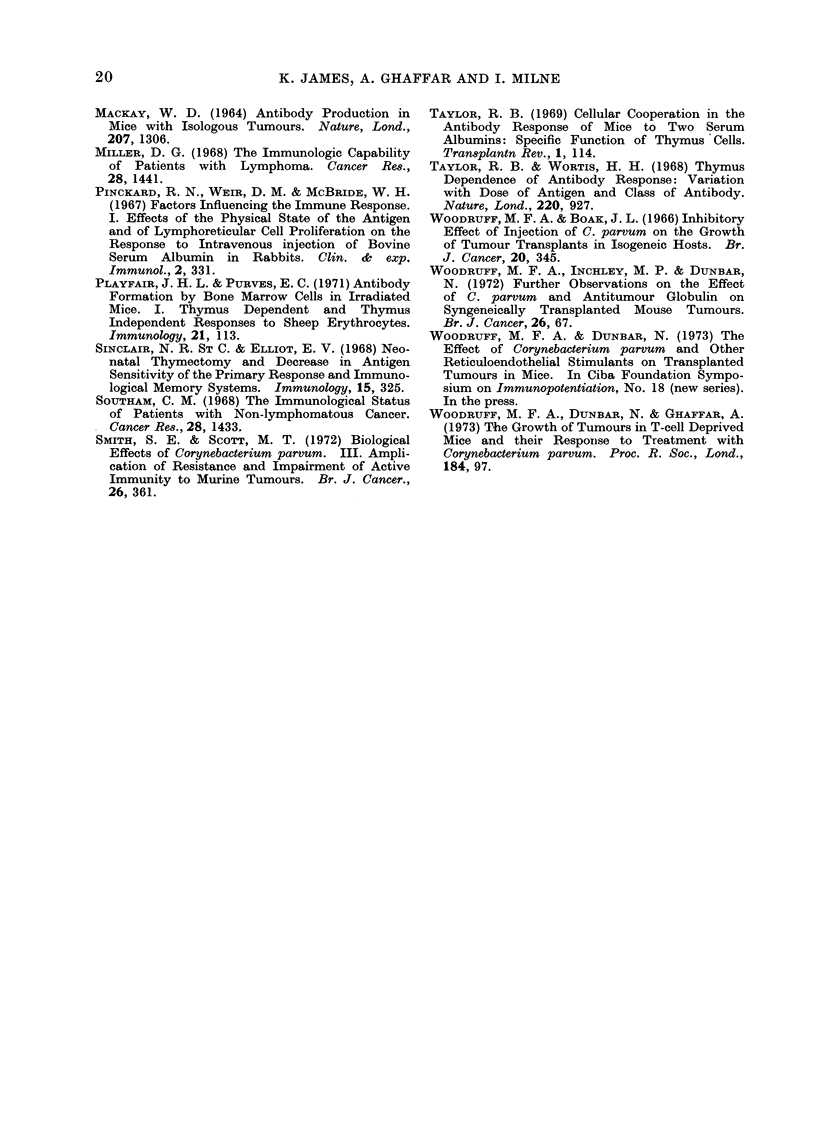


## References

[OCR_01429] Alexander P., Bensted J., Delorme E. J., Hall J. G., Hodgett J. (1969). The cellular immune response to primary sarcomata in rats. II. Abnormal responses of nodes draining the tumour.. Proc R Soc Lond B Biol Sci.

[OCR_01436] Brooks W. H., Netsky M. G., Normansell D. E., Horwitz D. A. (1972). Depressed cell-mediated immunity in patients with primary intracranial tumors. Characterization of a humoral immunosuppressive factor.. J Exp Med.

[OCR_01453] FARR R. S. (1958). A quantitative immunochemical measure of the primary interaction between I BSA and antibody.. J Infect Dis.

[OCR_01466] Ghaffar A., James K. (1973). The effect of antilymphocytic antibody on the humoral immune response in different strains of mice. 3. The response to type 3 pneumococcus polysaccharide.. Immunology.

[OCR_01473] Halpern B. N., Biozzi G., Stiffel C., Mouton D. (1966). Inhibition of tumour growth by administration of killed corynebacterium parvum.. Nature.

[OCR_01479] Howard J. G., Christie G. H., Courtenay B. M., Leuchars E., Davies A. J. (1971). Studies on immunological paralysis. VI. Thymic-independence of tolerance and immunity to type 3 pneumococcal polysaccharide.. Cell Immunol.

[OCR_01495] James K., Milne I. (1972). The effect of anti-lymphocytic antibody on the humoral immune response in different strains of mice. I. The response to bovine serum albumin.. Immunology.

[OCR_01502] Kamo I., Ishida N. (1971). Immune response in tumor-bearing mice.. Gan.

[OCR_01511] LESKOWITZ S., PHILLIPINO L., HENDRICK G., GRAHAM J. B. (1957). Immune response in patients with cancer.. Cancer.

[OCR_01516] LYTTON B., HUGHES L. E., FULTHORPE A. J. (1964). CIRCULATING ANTIBODY RESPONSE IN MALIGNANT DISEASE.. Lancet.

[OCR_01506] Lee A. K., Rowley M., Mackay I. R. (1970). Antibody-producing capacity in human cancer.. Br J Cancer.

[OCR_01523] Mackay W. D. (1965). Antibody production in mice with isologous tumours.. Nature.

[OCR_01528] Miller D. G. (1968). The immunologic capability of patients with lymphoma.. Cancer Res.

[OCR_01533] Pinckard R. N., Weir D. M., McBride W. H. (1967). Factors influencing the immune response. I. Effects of the physical state of the antigen and of lymphoreticular cell proliferation on the response to intravenous injection of bovine serum albumin in rabbits.. Clin Exp Immunol.

[OCR_01542] Playfair J. H., Purves E. C. (1971). Antibody formation by bone marrow cells in irradiated mice. I. Thymus-dependent and thymus-independent responses to sheep erythrocytes.. Immunology.

[OCR_01549] Sinclair N. R., Elliott E. V. (1968). Neonatal thymectomy and the decrease in antigen-sensitivity of the primary response and immunological "memory" systems.. Immunology.

[OCR_01560] Smith S. E., Scott M. T. (1972). Biological effects of Corynebacterium parvum. 3. Amplification of resistance and impairment of active immunity to murine tumours.. Br J Cancer.

[OCR_01555] Southam C. M. (1968). The immunologic status of patients with nonlymphomatous cancer.. Cancer Res.

[OCR_01567] Taylor R. B. (1969). Cellular cooperation in the antibody response of mice to two serum albumins: specific function of thymus cells.. Transplant Rev.

[OCR_01573] Taylor R. B., Wortis H. H. (1968). Thymus dependence of antibody response: variation with dose of antigen and class of antibody.. Nature.

[OCR_01579] Woodruff M. F., Boak J. L. (1966). Inhibitory effect of injection of Corynebacterium parvum on the growth of tumour transplants in isogenic hosts.. Br J Cancer.

[OCR_01585] Woodruff M. F., Inchley M. P., Dunbar N. (1972). Further observations on the effect of C. parvum and anti-tumour globulin on syngeneically transplanted mouse tumours.. Br J Cancer.

